# LINC00210 as a miR-328-5p sponge promotes nasopharyngeal carcinoma tumorigenesis by activating NOTCH3 pathway

**DOI:** 10.1042/BSR20181168

**Published:** 2018-11-16

**Authors:** Shu Zhang, Ping Li, Lei Zhao, Ling Xu

**Affiliations:** 1 Department of Otorhinolaryngology, Affiliated Hospital of Inner Mongolia Medical University, Hohhot 010050, China; 2 Department of Obstetrics, Yongkang Maternal and Child Health Care Hospital, Yongkang 321300, China; 3 Department of Radiology, Affiliated Hospital of Inner Mongolia Medical University, Hohhot 010050, China

**Keywords:** invasion, Long noncoding RNA, LINC00210, nasopharyngeal carcinoma, proliferation

## Abstract

As a kind of essential regulators, long noncoding RNAs (lncRNAs) have attracted a lot of attention in recent years. Nevertheless, the function of lncRNA in nasopharyngeal carcinoma (NPC) remains poorly understood. In the present study, we explained the role and mechanism of LINC00210 in NPC progression. We found that LINC00210 expression was up-regulated in NPC samples. Besides, its overexpression was positively correlated with NPC metastasis while predicting poor prognosis. Based on functional experiments, we revealed that LINC00210 contributed to NPC cell proliferation and invasion *in vitro*, and promotes tumor growth *in vivo*. Mechanistically, we identified that LINC00210 was located in the cytoplasm of NPC cells and served as the miR-328-5p sponge. Furthermore, we showed that miR-328-5p targets the 3′ untranslated region (3′-UTR) of NOTCH3. Through inhibiting miR-328-5p activity, LINC00210 promoted NOTCH3 expression in NPC, leading to activation of NOTCH3 signaling pathway. In conclusion, our study indicates LINC00210 promotes NPC progression through modulating proliferation and invasion.

## Introduction

Nasopharyngeal carcinoma (NPC), originated from the epithelial cells in nasopharynx, is the most prevalent and aggressive head and neck cancer worldwide [1]. In spite of much advancement on adjuvant chemotherapy and radiation strategy for NPC prevention, the 5-year overall survival rate remains still poor among NPC patients [2]. Notably, lymphoid node metastasis is usually observed in NPC, which greatly contributes to malignance and death [3]. Therefore, to develop effective strategies for NPC treatment, it is necessary to investigate the mechanism underlying NPC malignant behaviors such as proliferation and metastasis.

As reported, more than 95% of the human genomic transcripts belong to noncoding RNAs, including microRNA (miRNA), long noncoding RNA (lncRNA) and circular RNA (circRNA) [4]. Among them, lncRNA is characterized by a length of over 200 nucleotides and no or limited protein-coding ability [5–8]. Recently, lncRNA has been the research focus in biology. Growing studies show that lncRNA possesses critical functions in multiple processes, such as development, immune and cancer [9,10]. LncRNA is involved in regulation of proliferation, migration and cell death in tumor through modulating miRNA availability and gene expression [11]. For example, Lin et al. [12] reported that lncRNA1 sponges miR-885-3p to up-regulate CDK4 expression and consequently contributes to gastric cancer progression. Qu et al. [13] showed that lncRNA SOX2OT promotes gastric malignancy through attenuating miR-194-5p-induced inhibition on AKT2 expression. Additionally, Cheng and colleagues indicated that lncRNA XIST is up-regulated in NPC tissues and promotes tumor growth via inhibiting miR-491-5p [14]. Moreover, some evidences reveal lncRNAs might be biomarkers and therapeutic targets for cancer intervention [15]. However, the function of lncRNA in NPC still remains limitedly understood.

A recent study showed that LINC00210 promotes liver cancer development [16]. Whether LINC00210 plays some roles in NPC needs to be explored. In the present study, we sought to determine the correlation between LINC00210 and NPC progression. Our results showed that LINC00210 expression was positively correlated with NPC metastasis and progression. LINC00210 contributes to NPC cell proliferation and invasion dramatically. In mechanism, we revealed that LINC00210 activated NOTCH3 pathway through abolishing miR-328-5p-mediated inhibition. In collection, our study provides a novel insight on the mechanism of NPC progression.

## Materials and methods

### Sample tissues

Fifty-one histologically diagnosed NPC tissues and non-tumor tissues were included and obtained from Affiliated Hospital of Inner Mongolia Medical University. All tumor tissues and control tissues were validated by three independent pathologists before usage. Clinicopathological characteristics of these samples were contained in Table 1. The present study was approved by the Ethics Committee at Affiliated Hospital of Inner Mongolia Medical University. All written informed consents were signed by subjects.

**Table 1:** Association between LINC00210 expression and clinicopathological characteristics

Characteristics	INC00210 expression		*P*-value
	Low (*n*=31)	High (*n*=20)	
Gender			0.247
Male	18	15	
Female	13	5	
Age			0.267
<48	15	13	
≥48	16	7	
Clinical stages			0.042
I-II	21	7	
III-IV	10	13	
Metastasis			0.015
Yes	3	8	
No	28	12	

Pearson Chi-square test was used to calculated *P*-value.

### Cell culture and transfection

NPC cell lines (5-8F, 6-10B, S18 and S26) and immortalized nasopharyngeal epithelial cell line NP69 were maintained in RPMI-1640 medium containing 10% FBS (Gibico, Grand Island, U.S.A.) and cultured at a humidified atmosphere with 5% CO_2_. siRNA, miR-328-5p mimics (5′-GGGGGGGCAGGAGGGGCUCAGGG-3′), miR-328-5p inhibitors (5′-CCCTGAGCCCCTCCTGCCCCCCC-3′) and negative controls (5′-UCACAACCUCCUAGAAAGAGUAGA-3′) were purchased from Sangon Biotech (Shanghai, China). siRNAs against LINC00210 (5′-GGUUCUCAUUCUCAUUUAAUU-3′) or NOTCH3 (5′-GCUGGAUUCUGUGUACCUAGU-3′) and negative control (siNC, 5′-AAUUCUCCGAACGUGUCACGU-3′) were synthesized by RiboBio (Guangzhou, China). Cell transfection was performed using Lipofectamine 2000 (Invitrogen, Carlsbad, CA) as described before [17].

### qRT-PCR

Total RNAs were extracted using TRIzol reagent (Invitrogen, U.S.A.) from tissues or cell lines according to the manufacturer’s instructions. Complementary DNA (cDNA) was synthesized utilizing PrimeScript RT reagent Kit (Takara, Dalian China) following the manufacturer’s protocols. qRT-PCR was performed by using RealMasterMix (SYBR Green) (Tiangen) according to the manufacturer’s protocols. Gene expression was normalized to U6 or GAPDH and calculated according to 2^−ΔΔ*C*^_t_ method. Primer sequences were as follows: LINC00210 (Forward, 5′-AACACGTTAGCGGGTTCTCA-3′ and Reverse, 5′-TCAAAAACCACCGAGGGAGG-3′), miR-328-5p (Forward, 5′-AACGAGACGACGACAGAC-3′ and Reverse, 5′-GGGGGGGCAGGAGGGGCTCAGGG-3′), NOTCH3 (Forward, 5′-CAGTGTGAACTCCTCTCCCC-3′ and Reverse, 5′-GGTGCAGATACCATGAGGGC-3′), JAG1 (Forward, 5′-AATGGCTACCGGTGTGTCTG-3′ and Reverse, 5′-CCCATGGTGATGCAAGGTCT-3′), HES1 (Forward, 5′-CTGAGCACAGACCCAAGTGT-3′ and Reverse, 5′-GAGTGCGCACCTCGGTATTA-3′), HES6 (Forward, 5′-TGCGGCTGCTGCTGG-3′ and Reverse, 5′-TGCATGCACTGGATGTAGCC-3′), HEY1 (Forward, 5′-CGGCTCTAGGTTCCATGTCC-3′ and Reverse, 5′-GCTTAGCAGATCCTTGCTCCA-3′), HEY2 (Forward, 5′-GTGGGAAAGAGCCGCTAGG-3′ and Reverse, 5′-GTCTCGTCCATGTCGCTCTC-3′), CCND1 (Forward, 5′-TGAGGGACGCTTTGTCTGTC-3′ and Reverse, 5′-GCCTTTGGCCTCTCGATACA-3′), MYC (Forward, 5′-CCCTCCACTCGGAAGGACTA-3′ and Reverse, 5′-GCTGGTGCATTTTCGGTTGT-3′), U6 (Forward, 5′-AACGAGACGACGACAGAC-3′ and Reverse, 5′-GCAAATTCGTGAAGCGTTCCATA-3′) and GAPDH (Forward, 5′-ATGTTGCAACCGGGAAGGAA-3′ and Reverse, 5′-AGGAAAAGCATCACCCGGAG-3′).

### Cell proliferation analysis

For CCK8 assay, 2 × 10^3^ cells were plated in a 96-well plates and cultured for indicative hours. Then 10 μl CCK8 solution was added into 100 μl medium per well. After incubation for 1 h at 37°C, the absorbance at 450 nm was measured using a microplate reader. For colony formation assay, 3 × 10^2^ cells per well were seeded into a six-well plate and cultured for 12 days. Then the colonies were fixed using methanol and stained with 0.2% crystal violet, followed by photographing.

### Transwell invasion assay

Transwell invasion assay was performed according to a previous report [17]. In brief, 2 × 10^4^ cells were seeded in the upper chamber (pre-coated with Matrigel, Corning, Life sciences, U.S.A.) with 200 μl serum-free medium. About 600 μl of 10% FBS-containing medium was added in the lower chamber. After cultured for 1 day, the cells invading into the lower chamber were fixed with methanol and stained with 0.2% crystal violet, followed by photographing using an inverted microscope.

### Luciferase reporter assay

Dual luciferase reporter assay was performed as described previously [18].

### RNA pull-down assay

RNA pull-down assay was performed according to a previous study [4]. In brief, biotin-labeled LINC00210 or control was obtained by *in vitro* transcription using AmpliScribe T7-Flash Biotin-RNA Transcription Kit (Epicentre) according to the manufacturer’s instructions. Biotin-LINC00210 or NC was added into the NPC cell lysates as well as Streptavidin-coated Dynabeads and incubated for 6 h at 4°C. Then the beads were isolated, and precipitated RNAs were eluted and extracted, followed by qRT-PCR analysis.

### Xenograft experiment

Four-week-old male nude mice were purchased from Beijing Vital River Laboratory Animal Center. About 5 × 10^6^ WT or LINC00210 stably depleted 5-8F cells in 100 μl medium were subcutaneously injected into the flank of each nude mice. Tumor volume and weight were monitored at indicative days. This experiment was approved by the Animal Ethics Committee at Affiliated Hospital of Inner Mongolia Medical University .

### Statistical analysis

All results were analyzed utilizing GraphPad Prism or SPSS 23.0 software (SPSS, Chicago, IL, U.S.A.) and expressed as the means ± SD. The significance of difference was calculated using Student’s *t*-test or a one-way ANOVA. *P*<0.05 shows significant.

## Results

### LINC00210 up-regulation correlates with NPC progression

**Figure 1:**
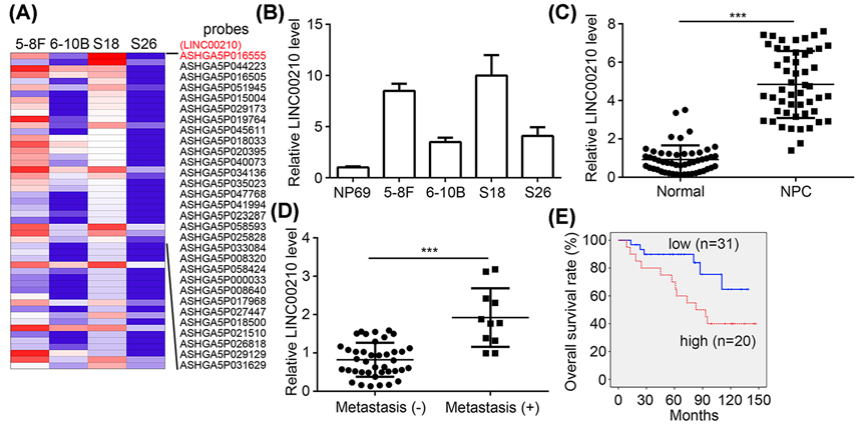
LINC00210 up-regulation correlates with NPC progression (**A**) The most up-regulated 50 lncRNAs in high-metastasis NPC cell lines (5-8F and S18) compared with low-metastasis cell lines (6-10B and S26). Red: high expression; Blue: low expression. (**B**) LINC00210 expression was up-regulated in NPC cell lines compared with NP69 cells. (**C**) LINC00210 levels were up-regulated in NPC tissues compared with adjacent normal tissues. (**D**) LINC00210 expression was higher in metastatic NPC tissues compared with non-metastatic tissues. (**E**) Higher expression of LINC00210 predicted lower overall survival rate in NPC patients; ***P<0.001.

Metastasis contributes to the malignance of NPC. Thus, we sought to search important lncRNAs involved in NPC metastasis. Through analyzing an online microarray cohort (GSE89804), we identified many differentially expressed lncRNAs (Figure 1A). Among them, LINC00210 was the most highly expressed lncRNA in high-metastasis NPC cell lines (5-8F and S18 cells) (Figure 1A). We then performed qRT-PCR and validated its high expression in NPC cell lines, including 5-8F and S18 cells (Figure 1B). To determine whether LINC00210 expression displays a similar trend in NPC tissues, we assessed its expression patterns. We found that LINC00210 expression was significantly elevated in NPC tissues compared with adjacent normal tissues (Figure 1C). Moreover, LINC00210 expression was higher in metastatic tissues than that in non-metastatic tissues (Figure 1D), which is consistent with the microarray result in Figure 1A. To further determine whether LINC00210 could act as a prognostic biomarker, we conducted Kaplan–Meier curve analysis according to LINC00210 expression in NPC tissues. The results revealed that higher LINC00210 expression correlates with lower survival rate (Figure 1E). In summary, LINC00210 might contribute to NPC progression.

### Knockdown of LINC00210 suppressed NPC growth and invasion

**Figure 2:**
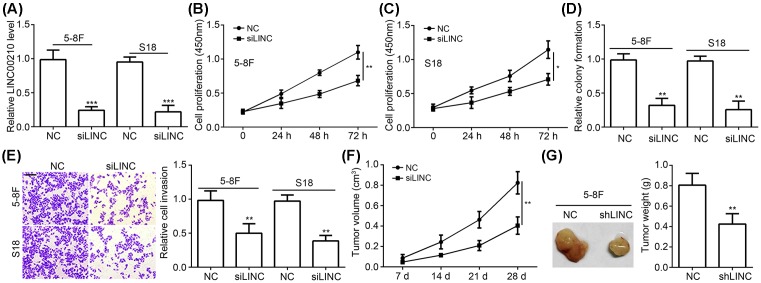
Knockdown of LINC00210 suppressed NPC growth and invasion (**A**) LINC00210 expression was dramatically decreased in 5-8F and S18 cells after si-LINC00210 transfection. (**B** and **C**) CCK8 results indicated that LINC00210 knockdown reduced cell proliferation potential. (**D**) Colony formation assay showed that LINC00210 depletion decreased formed colony number. (**E**) Transwell assay indicated that LINC00210 knockdown suppressed NPC cell invasion; scale bar: 100 μm. (**F** and **G**) LINC00210 knockdown significantly inhibited tumor volume and weight. 5-8F cells were injected into nude mice for Xenograft experiments; *P<0.05, **P<0.01 and ***P<0.001.

In order to identify the function of LINC00210, we chose the metastatic NPC cell lines (5-8F and S18) to perform experiments. We first silenced LINC00210 in 5-8F and S18 cells through siRNAs (Figure 2A). Then CCK8 and colony formation assays were conducted. The results indicated that LINC00210 silence led to attenuated proliferation and colony formation (Figure 2B–D). We have demonstrated LINC00210 expression positively correlated with metastasis. Thus, we performed Transwell assay to check the effect of LINC00210 on NPC cell metastasis. The results showed that LINC00210 knockdown significantly inhibited cell invasion (Figure 2E). In order to further confirm the role of LINC00210 *in vivo*, we conducted a Xenograft model. We monitored the tumor volumes and found that the volumes were smaller in LINC00210-depleted group (Figure 2F). Consistently, the tumor weights were lighter in LINC00210-silenced group compared with control group on day 28 post injection (Figure 2G). Collectively, above results indicated that LINC00210 promotes NPC growth and metastasis.

### LINC00210 as a miR-328-5p sponge promoted NPC cell proliferation and invasion

**Figure 3:**
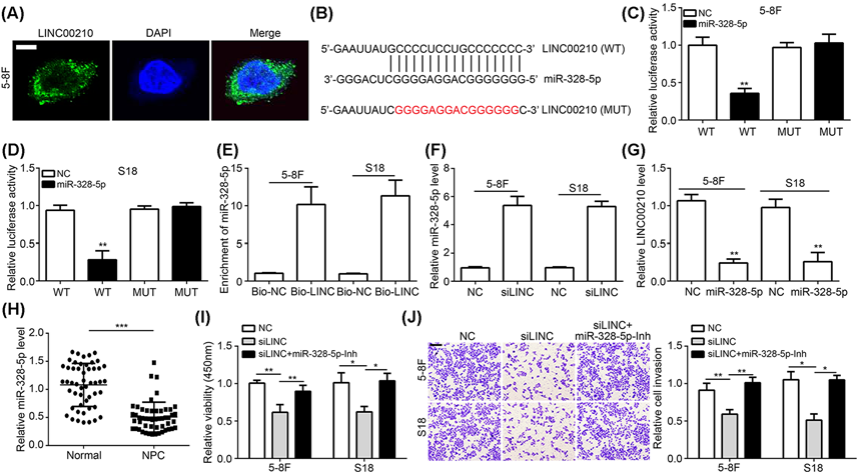
LINC00210 as a miR-328-5p sponge promoted NPC cell proliferation and invasion (**A**) RNA-FISH showed that LINC00210 mainly located in the cytoplasm of 5-8F cells; scale bar: 5 μm. (**B**) Predicted binding site for miR-328-5p in LINC00210 by bioinformatics analysis. (**C** and **D**) Luciferase reporter assay showed that miR-328-5p overexpression inhibited the luciferase activity of LINC00210-WT reporter in 5-8F and S18 cells. (**E**) Biotin-labeled LINC00210 precipitated miR-328-5p in 5-18F and S18 cells. (**F**) LINC00210 knockdown promoted miR-328-5p expression in 5-18F and S18 cells. (**G**) Overexpression of miR-328-5p inhibited LINC00210 expression in 5-18F and S18 cells. (**H**) Relative expression of miR-328-5p in NPC tissues by qRT-PCR. (**I**) CCK8 assay showed that miR-328-5p inhibition rescued the proliferation ability of LINC00210-depleted 5-18F and S18 cells. (**J**) Transwell assay showed that miR-328-5p inhibition rescued the invasive ability of LINC00210-depleted 5-18F and S18 cells; scale bar: 100 μm; *P<0.05, **P<0.01 and ***P<0.001.

As miRNA sponges to play roles, lncRNAs are proven to regulate tumorigenesis [19]. We determined the localization of LINC00210 in NPC cells and found that it mainly located in the cytoplasm (Figure 3A), suggesting LINC00210 might be a miRNA sponge. Through bioinformatics analysis (miRBase), we identified LINC00210 as a potential target of miR-328-5p (Figure 3B). To confirm it, luciferase reporter assay was conducted. Results showed that miR-328-5p overexpression decreased the activity of LINC00210-WT reporter in 5-8F and S18 cells (Figure 3C,D). Besides, biotin-labeled LINC00210 could precipitate miR-328-5p in NPC cell lysates (Figure 3E). These data demonstrated that LINC00210 directly interacts with miR-328-5p in NPC. Furthermore, qRT-PCR analysis indicated that LINC00210 silence promoted miR-328-5p expression, while miR-328-5p overexpression suppressed LINC00210 levels in 5-8F and S18 cells (Figure 3F,G). We also found that miR-328-5p expression was down-regulated in NPC tissues compared with normal tissues (Figure 3H).

After proving LINC00210 regulates miR-328-5p expression, we speculated that LINC00210 might play functions through miR-328-5p in NPC. To confirm this hypothesis, we conducted CCK8 and Transwell assays. We found that inhibition of miR-328-5p could significantly rescue the abilities of proliferation and invasion in 5-8F and S18 cells transfected with si-LINC00210 (Figure 3I,J).

### LINC00210 exerted roles through activating NOTCH3 pathway via sponging miR-328-5p

**Figure 4:**
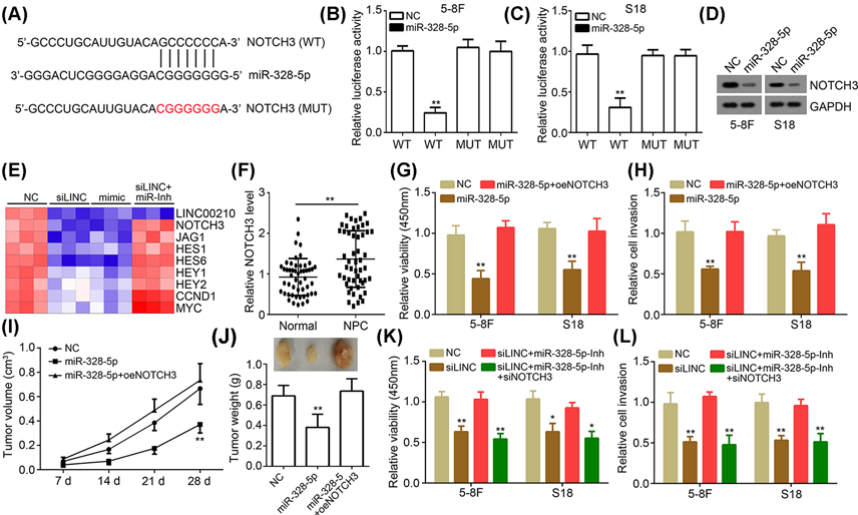
LINC00210 exerted roles through activating NOTCH3 pathway via sponging miR-328-5p (**A**) NOTCH3 was a predicted target of miR-328-5p. (**B** and **C**) Overexpression of miR-328-5p inhibited the luciferase activity of NOTCH3-WT reporter. (**D**) Western blotting for NOTCH3 expression in 5-18F and S18 cells transfected with described plasmids. (**E**) LINC00210/miR-328-5p axis regulated NOTCH3 pathway. The expression levels of LINC00210, NOTCH3, JAG1, HES1, HES6, HEY1, HEY2, CCND1 and MYC were analyzed by qRT-PCR in 5-8F cells. (**F**) NOTCH3 expression was up-regulated in NPC tissues. (**G** and **H**) CCK8 and Transwell assays showed that ectopic expression of NOTCH3 rescued miR-328-5p-mediated inhibition of NPC cell proliferation and invasion. (**I** and **J**) Xenograft assay showed that ectopic expression of NOTCH3 rescued miR-328-5p-mediated inhibition of NPC growth *in vivo*. (**K** and **L**) CCK8 and Transwell assays showed that LINC00210/miR-328-5/NOTCH3 pathway contributed to NPC cell proliferation and invasion; *P<0.05 and **P<0.01.

Afterward, we explored the target of miR-328-5p. Through bioinformatics analysis (TargetScan7), we identified NOTCH3 as a candidate (Figure 4A). Luciferase reporter assay showed that miR-328-5p overexpression suppressed the activity of NOTCH3-WT in 5-8F and S18 cells (Figure 4B,C), indicating miR-328-5p directly acts on NOTCH3. Western blotting showed that miR-328-5p mimics suppressed the expression of NOTCH3 in 5-8F and S18 cells (Figure 4D). Moreover, qRT-PCR analysis showed that both LINC00210 silence and miR-328-5p overexpression inhibited NOTCH3 expression and the activation of NOTCH3 pathway, as its target gene levels were significantly decreased (Figure 4E). However, inhibition of miR-328-5p abrogated the effects of LINC00210 knockdown (Figure 4E), suggesting LINC00210 promoted NOTCH3 pathway activation by inhibiting miR-328-5p. Furthermore, we found that the expression of NOTCH3 in NPC tissues was significantly increased compared with normal tissues (Figure 4F), implying its oncogenic role. We then performed rescue assays. Through CCK8 and Transwell assays, we found that ectopic expression of NOTCH3 restored miR-328-5p-mediated inhibition of NPC cell proliferation and invasion *in vitro* (Figure 4G,H). Moreover, Xenograft assay also demonstrated that ectopic expression of NOTCH3 restored miR-328-5p-mediated inhibition of NPC growth *in vivo* (Figure 4I,J), suggesting that decreased expression of NOTCH3 is involved in the effects of miR-328-5p.

To further determine whether LINC00210 exerts roles through regulating miR-328-5p/NOTCH3 pathway, we conducted CCK8 and Transwell assays. As shown, NOTCH3 knockdown significantly inhibited the proliferation and invasion of 5-8F and S18 cells transfected with both si-LINC00210 and miR-328-5p inhibitors (Figure 4K,L). Taken together, our study demonstrated that LINC00210 possesses oncogenic roles through regulating miR-328-5p/NOTCH3 pathway in NPC.

## Discussion

Most NPC-induced deaths are due to cancer recurrence and metastasis. Thus, it is critical to understand the mechanism underlying NPC progression. In the present study, we demonstrated that LINC00210 expression was up-regulated in NPC tissues and cell lines. Furthermore, the level of LINC00210 was correlated with NPC metastasis and prognosis in NPC. Knockdown of LINC00210 dramatically inhibited NPC cell proliferation and invasion *in vitro* and impaired cancer growth *in vivo*. This preliminary research indicated that LINC00210 might be a promising candidate for anti-metastasis therapy in NPC.

Accumulating studies have showed that lncRNAs exert vital biological functions in human cancers [20–23]. Dysregulated expression of lncRNAs often contributes to the development and progression of cancers [24]. In NPC, a few of lncRNAs have been identified as indicators for patients’ prognosis. For instance, Nie et al. [19] showed that lncRNA ZNF674-1 down-regulation predicts poor prognosis in NPC. Wu et al. [25] showed that lncRNA ANRIL is up-regulated by SOX2 and promotes the growth of NPC cells. Cheng et al. [26] reported that lncRNA NEAT1 modulates miR-124/NF-κB signaling to enhance NPC progression. Bo et al. [27] demonstrated that overexpressed AFAP1-AS1 promotes NPC growth and metastasis, predicts poor prognosis. However, the function of most lncRNAs in NPC still remains largely unknown. Although a recent study showed that LINC00210 promotes liver cancer progression [16]. Whether LINC00210 is implicated in other cancer requires investigation. Our study demonstrated that LINC00210 facilitates NPC progression, and correlates with tumor metastasis and prognosis.

Long RNAs are shown to sponge miRNAs in cancer [19,28,29]. We further investigated the target of LINC00210. Through bioinformatics method and functional experiments, we identified and demonstrated that LINC00210 interacted miR-328-5p to inhibit its expression in NPC cells. miRNAs also belong to noncoding RNAs and participate in various cancers [30–32]. A lot of studies indicate miR-328 as a tumor suppressor. For instance, Luo et al. [33] showed that miR-328-5p suppressed the growth of breast cancer through inhibiting RAGE. Wang et al. [34] showed that miR-328 suppressed cell proliferation and metastasis in cervical cancer via repressing TCF7L2. Han et al. [35] showed that miR-328 promotes cellular apoptosis in esophageal cancer through inhibiting PLCE1. In our study, we found that miR-328-5p inhibition promotes cellular proliferation and invasion in NPC, suggesting its tumor-suppressive function. We further screened out the downstream effector gene of miR-328-5p. We demonstrated that NOTCH3 was directly targeted by miR-328-5p. The expression of NOTCH3 was up-regulated by LINC00210-mediated inhibition on miR-328-5p. NOTCH3 was a key player in NOTCH signaling pathway, whose activation promotes expression of a series of oncogenes, such as MYC, HEY6 and CCND1 [36]. Previous reports also revealed that NOTCH3 promotes NPC growth and malignance [37]. However, the correlation between NOTCH3 and LINC00210 or miR-328-5p remains unclear. Our study for the first time demonstrated that NOTCH3 regulated by LINC00210/miR-328-5p axis contributes to NPC progression.

In summary, the present study provides evidence that LINC00210 modulating miR-328-5p/NOTCH3 pathway facilitates NPC progression. Our data suggested that LINC00210 might be a therapeutic target for NPC prevention. However, our work did not elucidate the mechanisms of LINC00210 induction and how LINC00210 expressions are regulated in normal and tumor tissues. Thus, it is necessary to investigate above problems in future.

## Abbreviations

lncRNA: long noncoding RNA
miRNA: microRNA
NPC: nasopharyngeal carcinoma
